# Special Session

**DOI:** 10.1111/ene.70232

**Published:** 2025-06-21

## Saturday, June 21 2025

## The description of neurological disorders in the arts

## SPS01_1

### Epilepsy and music

#### S. Evers

##### Department of Neurology, Krankenhaus Lindenbrunn, Münster, Germany

A link between epilepsy and music has already been proposed in the work of William Shakespeare. Since then, several citations have been found in the history of medicine. The first link is the provocation of epileptic seizures by music, that is, musicogenic epilepsy. This is regarded as a part of the reflex epilepsies. Clinical cases will be presented. The second link is music as a symptom of epileptic seizures. Here, the phenomenon of ictal singing and of aura continua musicalis have been described. The third link regards music as a treatment of epilepsy. The so‐called Mozart effect has been examined in this context: piano sonata KV 488 as a medicine against epilepsy in children. The fourth link is the influence of epilepsy and of the treatment of epilepsy on musical ability, It has been shown, for example, that untreated left temporal epilepsy impairs musical ability. The fifth link refers to epilepsy in famous composers and musicians. While in the history of music only little is known about composers with epilepsy (e.g., Mussorgsky, Gershwin), some famous artists in modern times use their epilepsy as a creative source for their music (e.g., Ian Curtis). Finally, a link between epilepsy and music can be seen in compositions. There are only examples of operas in which epileptic seizures are described such in Otello (Verdi) or Golem (d’Albert).

**Disclosure:** Nothing to disclose.

## SPS01_2

### Migraine in literature

#### M. Weatherall

##### Ealing Hospital, Department of Neurology, Southall, UK

## EAN/EPA: Brain and mental health across the lifespan: A neuropsychiatric perspective

## SPS02_1

### Adolescent brain development and early onset of psychiatric disorders

#### U. Volpe

##### Unit of Clinical Psychiatry, Department of Clinical Neurosciences/DIMSC, Università Politecnica delle Marche, Ancona, Italy

Adolescence marks a critical window in neurodevelopment characterized by profound structural and functional brain changes, particularly in regions governing emotion regulation, executive function, and social cognition. These neurobiological transitions coincide with a heightened vulnerability to the onset of psychiatric disorders, including mood, anxiety, psychotic, and substance use disorders. The interplay between normative brain maturation and the emergence of adult psychopathology is crucial for understanding the key neurodevelopmental trajectories and risk factors—genetic, environmental, and psychosocial—that contribute to early disease onset. Neuroimaging findings and longitudinal cohort studies will be examined to explore the implications of early identification and intervention strategies. By framing adolescent mental health within a lifespan neuropsychiatric perspective, this presentation underscores the need for integrative approaches to prevention and care during this formative period.

**Disclosure:** Nothing to disclose.

## SPS02_2

### Bridging neurology and psychiatry: The case for integrated brain health

#### P. Mohr

##### National Institute of Mental Health, Klecany, Czechia

## SPS02_3

### Brain Health starting from the beginning: The role of child neurology

#### G. Gröppel

##### Kepler University Hospital, Department of Paediatrics and Adolescent Medicine and Department for Neurology, Linz, Austria

## SPS02_4

### The Brain Health Mission, an inclusive platform for brain health advocacy and advancement in Europe

#### P. Boon

##### Ghent University Hospital, Department of Neurology, Ghent, Belgium

## Neurological disorders in Europe: Impact, costs, and the road ahead

## SPS12_1

### The burden of neurological diseases in Europe

#### M. Leone

##### IRCCS Casa Sollievo della Sofferenza, S. Giovanni Rotondo, Italy

Background: We estimate prevalence and health loss for 26 neurologic conditions across the WHO European Region from 2017 to 2021. Methods: We estimated mortality, prevalence, and disability‐adjusted life‐years (DALYs) by age and sex from the WHO Europe macroregion (East‐Central‐West Europe) for 2021, and for the 2017–2021 period. Findings: In 2021, an estimated 449.2 million (Mio) people in the WHO Europe had one or more diseases affecting the nervous system (50% of the population). The resulting disease burden was estimated to be 1.8 Mio deaths and 49.1 Mio DALYs. Globally, these 26 conditions were the most prevalent disease group the leading cause of death, and the second leading cause of for DALYs. Eight diseases accounted for more than 90% of DALYs in WHO Europe: stroke, Alzheimer's disease and other dementias, migraine, diabetic neuropathy, cancer of the nervous system, Parkinson's disease, epilepsy, and traumatic brain injury. The age‐standardised prevalence rate of all neurological diseases remained relatively stable from 2017 to 2021 (52.4 to 52.7 per 100,000, a 0.54% increase). The prevalence rates increased more than 5% for five conditions (neurosyphilis, diabetic neuropathy, GBS, tetanus, and cystic echinococcosis). Conversely, central nervous system cancer showed the largest decline (‐6.5%). In spite of a stable prevalence, age‐standardized death and DALYs rates showed a remarkable reduction of 6.0%, and of 2.5%. Interpretations: Neurological disorders are the leading cause of overall disease burden in Europe. Health authorities should prioritize neurological disease prevention and care.

**Disclosure:** Nothing to disclose.

## SPS12_2

### The cost of neurological disorders in Europe – COIN‐Eu

#### M. Montes‐Martinez ^1^, L. Welter ^1^, R. Dodel^1^, P. Boon^2^, C. Bassetti^3^, T. Berger^4^, E. Moro^5^, C. Kruse^1^, M. Lolich^6^, M. Konti^6^, M. Arvandi^7^, N. Mühlberger^7^, U. Siebert^7^

##### ^1^Department of Geriatric Medicine, University Duisburg‐Essen, Germany; ^2^Department of Neurology, 4Brain Ghent University Hospital & Ghent University, Ghent, Belgium; ^3^Medical Faculty, University of Bern, Neurology Department, Inselspital, Bern, Switzerland; ^4^Department of Neurology, Medical University of Vienna, Vienna, Austria; ^5^Grenoble Alpes University, CHU of Grenoble, Division of Neurology, Grenoble Institute of Neurosciences, Grenoble, France; ^6^European Academy of Neurology, Vienna, Austria; ^7^Institute of Public Health, Medical Decision Making and Health Technology Assessment, Department of Public Health and Health Technology Assessment, UMIT TIROL‐ University for Health Sciences and Technology, Hall in Tirol, Austria

Introduction: Neurological conditions are a leading global cause of health loss, accounting for 11.1 million deaths and 443 million disability‐adjusted life years. We aimed to estimate their macroeconomic societal burden across 12 disease groups in 47 European countries. Methods: We applied a four‐step approach: (1) epidemiological data were sourced from the Global Burden of Disease (GBD) Study 2021 and supplemented with literature reviews for selected conditions; (2) economic data were identified via systematic review; (3) data were extracted, pooled, and structured by country‐economic parameters, which were also used to impute values for countries lacking primary data; and (4) neurology experts assessed the plausibility of country‐level results. Costs were assessed from a societal perspective, including direct, indirect, and informal care, and reported at country and per‐patient levels. Results: Our findings point to total costs of approximately €1,232 billion in Europe in 2019 (adjusted for purchasing power parity) associated with 10 neurological disease groups. For sleep disorders and polyneuropathies, based on alternative epidemiological sources, we suggest additional costs of around €423 billion and €39 billion, respectively. Costs ranged from €149 million (meningitis) to €815 billion (headache disorders). Indirect costs comprised 42% of the total burden, followed by direct costs (37%) and informal care (21%). Conclusion: Our results indicate a substantial economic burden from neurological diseases in Europe, highlighting the need for better understanding, research, and more effective strategies—including health promotion, prevention, treatment, and rehabilitation—and targeted policy action. Data scarcity limits current analyses, especially in middle‐income regions.

**Disclosure:** Commissioned by the European Academy of Neurology, with additional support from the Lundbeck International Neuroscience Foundation.

## SPS12_3

### Costs and epidemiology of sleep disorders in Europe

#### C. Bassetti

##### Department of Neurology, Inselspital, University of Bern, Bern, Switzerland

## SPS12_4

### The future of general neurology

#### C. Bassetti

##### Department of Neurology, Inselspital, University of Bern, Bern, Switzerland

## Sunday, June 22 2025

## What's new on EAN guidelines?

## SPS04_1

### From the oven to the table: A scoping review on the roadmap for implementation of neurological guidelines

#### K. Rukavina

##### Hospital for Movement Disorders Beelitz, Beelitz, Germany

The European Academy of Neurology (EAN) strives to harmonize neurological care across Europe through the development of high‐quality clinical practice guidelines (CPGs), but their impact relies on effective implementation. We reviewed recent literature on CPG implementation in neurology and synthesized findings from 36 relevant studies. This lecture explores the challenges of CPG implementation, tackles common barriers and facilitators at the individual, organizational, and system levels, and proposes tailored planning approaches and targeted interventions to better translate EAN CPGs into everyday clinical practice.

**Disclosure:** Nothing to disclose.

## SPS04_2

### EAN‐MDS‐ES guideline on Parkinson's disease—Part II: Pharmacological management of motor symptoms—Section 1: First‐line treatment for initial monotherapy in early Parkinson's disease

#### K. Smilowska

##### Department of Neurology, Regional Hospital, Sosnowiec, Poland

Background The treatment options for early Parkinson's disease (PD) continues to evolve. In response, the European Academy of Neurology (EAN) and the Movement Disorder Society European Section (MDS‐ES) initiated a collaborative project to update clinical practice guidelines. Objective To provide updated, evidence‐based guidance tailored to the European context on the initiation of pharmacological therapy for motor symptoms in individuals with early PD. Methods A multidisciplinary panel of 16 neurologists and two patient representatives was convened by EAN and MDS‐ES. The panel adopted a methodology (GRADE‐ADOLOPMENT), which integrates elements of adoption, adaptation, and de novo development of recommendations, structured through GRADE Evidence to Decision frameworks. Two key review questions were established: the comparative efficacy of initial drug therapies and the risk of impulse control disorders associated with these treatments. Existing guidelines were systematically evaluated, and an updated systematic literature search was performed in March 2023 to identify relevant new clinical trial data. Results Two foundational guidelines—NICE (2017) and AAN (2021)—were selected as the basis for the evidence review. The panel reviewed recent evidence and synthesized findings related to available pharmacological interventions for early PD treatment. Focus was given to the balance of benefits and harms, patient values and preferences, and the feasibility and acceptability of each treatment approach. Conclusions This guideline project provides an updated synthesis of the current evidence for initiating pharmacological treatment in early PD. It aims to support more consistent, evidence‐based decision‐making across Europe, enhance patient‐clinician discussions, and improve overall care quality in the early Parkinson's disease.

**Disclosure:** KS has received honoraria from the European Academy of Neurology, International Parkinson and Movement Disorder Society, AbbVie and Pfizer.

## SPS04_3

### EAN‐ESCMID guidelines on the diagnosis and management of encephalitis in adults caused by infection

#### J. Sellner

##### Landesklinikum Mistelbach‐Gänserndorf, affiliated with Karl Landsteiner University of Health Sciences, Krems, Austria

Background Encephalitis, a life‐threatening condition associated with significant morbidity and mortality, is difficult to diagnose and manage. The latest European guidelines were published in 2010. Since this time, novel causes of encephalitis have emerged and diagnostic processes have evolved, which need to be reflected in updated guidelines. Objective We aimed to produce recommendations for the diagnosis and management of infectious encephalitis in adults based on current evidence and expert opinion to optimise diagnosis and treatment and improve patient outcome. Methods This guideline was developed by a multidisciplinary task force of 19 specialists, and recommendations were developed according to the GRADE approach. Where there was a lack of evidence or evidence was too indirect or heterogeneous, a good practice statement was made. Sixteen PICO questions were considered. Results The following two recommendations were reached: for initiation of intravenous acyclovir as soon as possible upon suspicion of encephalitis and against replacing pathogen‐specific polymerase chain reaction (PCR) testing of cerebrospinal fluid (CSF) with multiplex CSF PCR panels for diagnosis of adult patients with suspected infectious encephalitis. A further 14 good practice statements regarding when to clinically suspect infectious encephalitis, microbiological investigations, additional investigations, treatment, and ongoing care and rehabilitation were agreed. Conclusions Given the lack of direct evidence identified by this review, future large‐scale multi‐centre studies of encephalitis are encouraged. In particular, research should focus on novel treatments and diagnostics and development of a core outcome set definition for use in future trials of interventions for acute care and post‐encephalitis rehabilitation.

**Disclosure:** Nothing to disclose.

## SPS04_4

### EAN CoCoCare Graduation 2024

#### M. Majoie

##### Academic Centre for Epileptology, Epilepsy Centre Kempenhaeghe & Maastricht University Medical Centre, Netherlands

EAN CoCoCare Graduation 2024: Building Guideline Development Skills for Cost‐Conscious Neurological Care Clinical practice guidelines are essential tools in achieving high‐quality, cost‐efficient healthcare. Recognizing the need to strengthen competencies in this area, the Cost‐Conscious Healthcare (CoCoCare) training programme was initiated in 2018 at Maastricht University (Netherlands) and later adopted by the European Academy of Neurology (EAN). Initially supported by an EU Erasmus grant, the programme became an official EAN initiative in 2023. CoCoCare is a structured, year‐long educational programme aimed at neurology residents and early‐career neurologists. It equips participants with the skills needed to develop and implement evidence‐based, economically sound clinical guidelines. The training combines e‐learning, an in‐person kick‐off workshop at the EAN Congress, monthly webinars, self‐directed learning, and on‐the‐job practical guideline development. The course concludes with project presentations at the EAN Congress, providing both academic recognition and practical exposure. Since its launch, 114 participants from 23 countries have taken part. Feedback has highlighted the programme's impact on fostering critical reflection on cost‐conscious decision‐making and stimulating active involvement in guideline development. With its third edition launching at the EAN Congress 2025, CoCoCare continues to invest in the future of neurology by nurturing a new generation of experts capable of leading guideline development within the EAN framework. Graduates benefit from mentorship, congress participation grants, and enhanced professional visibility—contributing to the long‐term quality and sustainability of neurological care across Europe.

**Disclosure:** Nothing to disclose.

## Breakthroughs in treatment Neurology—Part 1

## SPS05_1

### Directly isolated allogeneic virus‐specific T cells in progressive multifocal leukoencephalopathy

#### L. Grote‐Levi

##### Department of Neurology, Hannover Medical School, Hannover, Germany

Progressive multifocal leukoencephalopathy (PML) is a life‐threatening viral brain infection that predominantly affects immunocompromised individuals. The primary therapeutic goal—reconstitution of the compromised immune system—is not always achievable, depending on the underlying condition. To date, no approved antiviral treatment is available. Treatment with directly isolated allogeneic virus‐specific (DIAVIS) T cells demonstrated promising therapeutic effects in 28 patients treated monocentric at the Hannover Medical School, between March 2020 and February 2022. In this retrospective case series, patients with definite, progressive PML received DIAVIS T cells as a single fresh infusion containing up to 2 × 10^4^ CD3^+^ cells/kg body weight. Remaining T cells were cryopreserved in divided aliquots and administered in additional doses approximately 2 and 6 weeks later. DIAVIS T cells, directed against the BK polyomavirus, were isolated from healthy donors within 24 hours. Twenty‐two patients (79%) showed a clinical response, characterized by stabilization or improvement of neurological symptoms and reduction in viral load. Six patients (21%) were classified as non‐responders and experienced rapid deterioration leading to death; two additional patients died during the 12‐month follow‐up period. Older age emerged as the only predictor of poor treatment response. Survival analysis demonstrated improved 12‐month survival rates for DIAVIS‐treated patients (18 of 26 [69%]; hazard ratio 0.42, 95% CI 0.24–0.73, *p* = 0.02) compared with historical controls receiving best supportive treatment (57 of 113 [50%]; 12‐month survival including censored data: 45%). This case series provides first class IV evidence that DIAVIS T‐cell therapy may reduce mortality and improve functional outcomes in patients with PML.

**Disclosure:** Lea Grote‐Levi reported receiving financial support from Deutsche Forschungsgemeinschaft (DFG, German Research Foundation) Young Academy – PRACTIS (Program of Hannover Medical School for Clinician Scientists; ID 413617135)

## SPS05_2

### Cipaglucosidase alfa plus miglustat in adults with late‐onset Pompe disease: A phase III open‐label extension study

#### A. Toscano

##### Azienda Ospedaliera Universitaria Gaetano Martino, Department of Clinical and Experimental Medicine, University of Messina, Messina, Italy

## SPS05_3

### Phase 2 proof‐of‐concept: Targeting PACAP pathway for migraine treatment

#### M. Ashina

##### Department of Neurology, Danish Headache Center, Copenhagen University Hospital‐Rigshospitalet, Copenhagen, Denmark

Pituitary adenylate cyclase–activating polypeptide (PACAP) and its receptors are expressed in migraine‐relevant structures, including the trigeminal ganglion, cranial vasculature, and sphenopalatine ganglion, underscoring PACAP's role in migraine initiation. Monoclonal antibodies against PACAP have been shown to block PACAP38‐induced cranial artery dilation and reduce headache in healthy human subjects. In a phase II randomized, double‐blind, placebo‐controlled trial, a single 750 mg infusion of Lu AG09222, a humanized monoclonal antibody targeting PACAP, was administered to adults with migraine who had failed two to four prior preventive therapies. Over the first four weeks post‐infusion, Lu AG09222 reduced mean monthly migraine days by 6.2 compared with 4.2 for placebo (difference, −2.0 days; 95% CI, −3.8 to −0.3; *p* = 0.02). Treatment was generally well tolerated, with adverse events occurring at rates similar to placebo. These data establish PACAP ligand neutralization as a compelling proof‐of‐concept approach for migraine prevention and support further evaluation in larger, longer‐term studies.

**Disclosure:** MA is a consultant, speaker, or scientific advisor for AbbVie, Astra Zeneca, Eli Lilly, GlaxoSmithKline, Lundbeck, Pfizer, and Teva; a primary investigator for ongoing AbbVie, Lundbeck and Pfizer trials. MA reports research grants from Lundbeck Foundation and Novo Nordisk Foundation (all to institution). MA serves as associate editor of the Journal of Headache and Pain and associate editor of Brain.

## SPS05_4

### CD19 CAR T therapy in myositis: Hype or hope?

#### M. de Visser

##### Academic Medical Centre Amsterdam, Department of Neurology, Amsterdam, The Netherlands

## SPS05_5

### Safety and efficacy of ocrelizumab in treatment of pediatric Multiple sclerosis

#### L. Papetti

##### IRCSS Bambino Gesù, Rome, Italy

## SPS05_6

### Efficacy and safety of elamipretide in individuals with Primary Mitochondrial Myopathy of nuclear origin

#### M. Mancuso

##### Azienda Ospedaliera Universitaria Pisana U.O.C. Neurologia, Pisa, Italy

Clinical Grand Round: Unravelling interesting neurological diagnoses

## SPS06_1

### Severe progressive tetraparesis out of the blue

#### L. Väli

##### Tartu University Hospital, Tartu, Estonia

A 56‐year‐old male presented with progressive leg weakness, hypesthesia, and upper body pain. EMG indicated acute demyelinating polyneuropathy. Despite initial treatments (IVIG, plasma exchange, corticosteroids), the condition worsened to severe flaccid tetraplegia over three months. Rituximab proved more effective. The patient is now ambulant with some restrictions and weak to moderate tetraparesis.

**Disclosure:** Nothing to disclose.

## SPS06_2

### Rare movement disorder, diagnosed by patient himself

#### K. Resik

##### Tartu University Hospital, Neurology Clinic, Tartu, Estonia

Abstract: An 18‐year‐old male patient presented to our epilepsy clinic with episodes of involuntary movements, which started at the age of four. These episodes were characterised by dancelike and writhing movements on one or both sides of the body and face. Episodes occurred 10 to 80 times a day with each lasting about 20 seconds. Awareness was fully retained. Episodes were triggered by voluntary activities, and he was able to induce an episode with sudden movements. The patient used Google and ChatGPT to find a diagnosis that explained his symptoms.

**Disclosure:** Nothing to disclose.

## SPS06_3

### Paroxysmal visual symptoms

#### K. Orav

##### Neurology Department, North Estonia Medical Centre, Tallinn, Estonia

A 27‐year‐old female reports episodes of flickering in the right eye with a sensation of right eye lateral deviation. During these attacks, other people's speech fades away and she can experience nausea. Headache may follow. These episodes started when she was 10 years old and currently occur almost daily. They usually last a few seconds, but vision can be impaired for hours. The attacks can be provoked by stressful situations. Additionally, the patient has separate episodes of retrograde amnesia lasting 24 hours which occur every 3 months.

**Disclosure:** Nothing to disclose.

## EAN/ESC: Neurology & cardiology meet epilepsy and critical care

## SPS07_1

### Heart‐brain‐interaction in cardiovascular disease

#### T. Lüscher

##### Zurich Heart House, Zurich, Switzerland

The heart and the brain interact extensively within the body. The two organs are connected by neural pathways, such as the sympathetic nervous system and the vagal nerve, regulating heart rate and cardiac contractability as well as vasomotor tone of the coronary circulation via beta‐and alpha‐adrenergic receptors. Furthermore, the heart feeds back signal into the brain, particularly through pain pathways, reaching the thalamus and the frontal cortex during episodes of ischaemia, leading to the perception of angina. Thus, the neural pathways affecting the brain and the heart are closely interconnected, in particular with the midbrain, such as the amygdala and hippocampus, thalamus and other centres.

A classic example of a cardiac disease that is basically a neurological condition with the heart as a target organ is the Takotsubo syndrome, mainly effecting post‐menopausal women. Our research has shown that appropriate processing of physical and psychological stimuli entering into the amygdala and hippocampus leads, due to inappropriate signal processing, to an overactivation of the sympathetic nervous system with a search of catecholamines and also endothelin leading to an increased vascular resistance, ischaemia with chest pain and eventually left ventricular dysfunction in the form of apical ballooning, mid‐ventricular, basal or antero‐lateral wall motion abnormalities. Although commonly transient in nature, the Takotsubo Syndrome is associated with significant complications, such as ventricular tachycardia and fibrillation, cardiogenic shock and cardiovascular death.

Similarly, the activity of the activity of the amygdala determines outcomes in patients with coronary artery disease. The activity of this mid‐brain structure, as assessed by 18Flurodeoxyglucose positron emission tomography, is predictive of a major cardiovascular events during long‐term following up suggesting that anxienty and other emotions do impact on the heart leading to increased major cardiovascular events.

Thus, neurophysiological research has markedly improved our understanding of the interaction of brain structures with the heart and the possible involvement of psychological and neurogenic factors in cardiovascular disease.

**Disclosure:** Nothing to disclose.

## SPS07_2

### Sudden unexpected death in epilepsy (SUDEP)

#### P. Ryvlin

##### Centre Hospitalier Universitaire Vaudois (CHUV), Lausanne, Switzerland

## SPS07_4

### Hypothermia and brain protection after cardiac arrest ‐ neurologic perspective

#### T. Cronberg

##### Lund University Hospital, Department of Neurology, Lund, Sweden

A cardiac arrest is typically caused by myocardial ischemia and it is a leading cause of death. Patients who are resuscitated usually remain unconscious on arrival and most will be transferred to an intensive care unit where approximately half will die during the coming week, most after a decision to withdraw intensive care based on a presumption of a poor neurological prognosis for meaningful recovery.

Whole body cooling before the onset of circulatory arrest will mitigate most pathological processes and has undisputable brain protective effects in experimental models and in numerous case reports of cold‐water drowning where survival with good outcome has been reported after >2 hours cardiac arrest with body temperature as low as 13°C. This protective effect is still used in thoracic surgery.

In 2002, two clinical trials were published, presenting evidence that systemic hypothermia after a cardiac arrest lead to increased survival and neurological function. This started an era of optimism for a new treatment strategy with potential also in traumatic brain injury and stroke. Implementation was rapid as was the development of new invasive and non‐invasive technology to cool. However, large trials with strictly controlled neuroprognostication and long‐term follow‐up have failed to show any difference in outcome between patients treated with 33 versus 36°C (TTM‐trial) and 33°C versus normothermia with fever prevention (TTM2‐trial). Accordingly, international guidelines have been updated to recommend fever prevention only.

This lecture will discuss current state of the art and remaining knowledge gaps for hypothermia as a treatment strategy for cardiac arrest victims.

**Disclosure:** Nothing to disclose.

## SPS10_1

### Acute and chronic stress, its complications, and the benefits of relaxation methods

#### M. Hilz

##### University of Erlangen‐Nuremberg, Germany, and Icahn School of Medicine at Mt. Sinai, New York, USA

Stress imperils physical and mental health. Hans Selye coined the term “Stress” and described it as “nonspecific response of an organism to any noxious or aversive event”. He distinguished chronic stress responses from the acute stress response that was first described by Walter Cannon as “Fight‐or‐Flight‐Response”. Selye recognized that chronic stress induces a response pattern that he called “General‐Adaptation‐Syndrome”: after an initial “alarm‐phase”, the organism tries to maintain homeostasis by activating coping strategies. After this “resistance‐phase” follows the “exhaustion‐phase”. Now, the organism is prone to disease or death. In response to stress, the so‐called “central stress system” mediates the “stress‐syndrome”, a range of responses that imply interactions of the central nervous system with endocrine pathways and the immune system. The “central stress system” is intertwined with the central autonomic network and activates the hypothalamic‐pituitary‐adrenocortical axis. Stress‐induced adrenaline release stimulates the renin‐angiotensin‐aldosterone system. Arginine‐vasopressin release increases renal water retention and affects blood pressure. The “stress‐syndrome” further involves catecholamine‐associated inflammasome upregulation. While sympathetic outflow increases, parasympathetic activity and its effects on organs as well as vagus‐mediated anti‐inflammatory effects decrease. Acute stressors may trigger syncope, arrhythmias, coronary artery occlusion, sudden death, Takotsubo syndrome, hypertensive crises, fear, panic attacks, etc. Chronic stress sequelae include obesity, diabetes, arterial hypertension, renal failure, stroke, myocardial infarction, pain syndromes, gastrointestinal, sexual, or cognitive dysfunction, depression, fatigue, burn‐out syndrome, pseudo‐dementia, etc. Endurance training, breathing techniques, Yoga, Tai‐Chi, meditation, prayer, olfactory stimuli, music, progressive muscle‐relaxation, functional relaxation, or autogenic training mitigate negative stress effects.

**Disclosure:** Related to this presentation I have nothing to disclose. I received honoraria for lecturing from Sanofi and travel support from Sanofi and Amicus Therapeutics.

## SPS10_2

### What is autogenic training?

#### M. Hilz

##### University of Erlangen‐Nuremberg, Germany, and Icahn School of Medicine at Mt. Sinai, New York, USA

Autogenic Training (AT) is a relaxation technique developed by the German psychiatrist Johannes Heinrich Schultz (1884–1970) and published in 1932. AT is widely used to counterbalance the negative effects of an acute or chronic “Stress‐syndrome”. In contrast to many relaxation techniques, AT yields “self‐generated” relaxation. Different from mediation techniques that use and repeat mantras, or from Progressive Muscle Relaxation which induces relaxation indirectly via voluntary repetitive contractions and relaxation of specific muscle groups, AT generates relaxation via self‐ or auto‐suggestion and passive, mental focusing on bodily perceptions, such as heaviness and warmth of a limb, sensations that in turn induce mental and physical relaxation with inner calm and tranquility. Persons practicing AT silently repeat a set of formulas that suggest and predict sensations that very likely occur regularly during AT, such as heaviness or warmth of a limb or perceiving free, automatic, slow respiration. Perceiving these sensations and breathing slowly and deeply augment parasympathetic and attenuate sympathetic outflow and thus mitigate or counterbalance the detrimental effects of acute or chronic stress. Electroencephalogram recordings during AT show increased alpha activity and reduced theta activity which indicates that the trainee is not dozing or sleeping but alert. Regularly practicing AT has shown beneficial effects in multiple somatic and mental disorders, including, for example, headaches, arterial hypertension, coronary artery disease, preeclampsia, asthma, pain disorders, functional sleep disorders, anxiety, depression, dysthymia, sexual arousal dysfunction, impaired memory or concentration. AT should not be used by psychotic persons.

**Disclosure:** I have nothing to disclose related to this presentation. I received lecturing honoraria from Sanofi and travel support from Sanofi and Amicus Therapeutics.

## SPS10_3

### Condensed introductory course of autogenic training

#### M. Hilz

##### University of Erlangen‐Nuremberg, Germany, and Icahn School of Medicine at Mt. Sinai, New York, USA

Autogenic Training (AT) uses formulas that suggest – and predict – sensations that subsequently manifest in most AT‐trainees, such as heaviness or warmth of limbs. While the trainee mentally repeats phrases that predict a sensation, the incipient, actual perception of this sensation reinforces the prediction as factual experience and thus corroborates the trainee's expectation and confidence that the self‐suggestions will indeed yield the predicted sensations that reflect and further relaxation. Hence, AT is based on observations of physiological phenomena and does not impose any external values, philosophies, or esoteric beliefs on trainees. The self‐suggestive formulas aim at perceiving and enhancing sensations of muscular relaxation sensed as heaviness of limbs, improved limb perfusion sensed as perception of warmth, stable cardiac function and rhythm perceived as regular, calm, and stable heart‐beat, enhanced, steady and unobstructed respiration perceived as automatic, free, and comforting regular breathing, increased splanchnic and bowel perfusion and function perceived as abdominal or epigastric warmth, and comfortable temperature or perfusion of the (fore)‐head perceived as “comfortable coolness of the forehead”. The very first self‐suggestion seems difficult in stressful situations but is effective: the trainee self‐suggests tranquility and calm to reach a state of focused silence and concentration. “I will be calm”, “nothing will disturb me” help achieve a state that facilitates perceiving the sensations predicted and induced by the aforementioned self‐suggestions. AT should be practiced regularly. Initially, two or three daily 20‐minute sessions might be needed for several weeks to achieve all self‐suggested effects of AT.

**Disclosure:** I have nothing to disclose related to this presentation. I received lecturing honoraria from Sanofi and travel support from Sanofi and Amicus Therapeutics.

## Monday, June 23 2025

## European Journal of Neurology: Clinical research that changed practice

## SPS08_1

### Pathophysiological underpinnings of recanalization therapies

#### J. Kõrv

##### Department of Neurology and Neurosurgery, University of Tartu, Tartu, Estonia

Stroke is the second‐leading cause of death and the third‐leading cause of combined death and disability worldwide. Acute ischemic stroke results from the occlusion of cerebral arteries, leading to a cascade of events including energy failure, excitotoxicity, and oxidative stress. Recanalization therapies, such as intravenous thrombolysis (IVT) and endovascular thrombectomy (EVT), aim to restore blood flow and mitigate these deleterious processes. Alteplase was the first thrombolytic used in the treatment of acute ischemic stroke, but it is increasingly being replaced by tenecteplase, a genetically modified tissue plasminogen activator with potentially superior efficacy in large vessel recanalization and practical workflow advantages. EVT, in addition to IVT, has been proven beneficial over IVT alone in patients with large vessel occlusions. However, outcomes are not always favorable even when successful recanalization of the occluded blood vessel is achieved. While successful recanalization is necessary for reperfusion, it does not guarantee it. The underlying pathophysiological mechanisms for recanalization and reperfusion are complex and multifaceted, involving molecular and cellular processes. Several mechanisms can cause tissue damage even if successful reperfusion is achieved. These include incomplete reperfusion/microvascular obstruction, blood‐brain barrier breakdown with resulting hemorrhagic complications and inflammatory changes, reperfusion‐ and excitotoxicity‐related injury, and secondary changes post‐infarction, including brain atrophy. Understanding these mechanisms is crucial for developing strategies to enhance the efficacy of recanalization therapies. Several promising neuroprotectant candidates as adjunctive treatments to recanalization therapies have been identified to further improve patient outcomes by preventing post‐recanalization tissue damage; however, none have been approved for clinical use yet.

**Disclosure:** Nothing to disclose.

## SPS08_2

### Autoimmune nodopathies

#### J. Devaux

##### Institut de Génomique Fonctionnelle, Université de Montpellier, CNRS, INSERM, Montpellier, France

Autoimmune nodopathy (AN) is a rare neuromuscular disorder affecting the nodes of Ranvier of myelinated axons in peripheral nervous system. Insofar, four pathogenic autoantibodies have been associated with AN and are good diagnostic and prognostic biomarkers. These autoantibodies target cell adhesion molecules which play crucial role in the function and formation of the node of Ranvier and of the paranodal region: neurofascin‐155 (Nfasc155), neurofascin‐186 (Nfasc186), contactin‐1 (CNTN1) and anti‐contactin associated protein 1 (CASPR1). Autoantibodies in AN are mainly of the IgG4 isotype. Since 2021, AN has been recognized as a separate pathological entity from chronic inflammatory demyelinating polyneuropathy (CIDP) due to the presence of these well‐described pathogenic antibodies and a specific response to therapeutic strategies. In this presentation, we will outline the clinical presentations and therapeutic responses of AN, and the specificities associated with each autoantibody. We will also recapitulate the knowledge on the pathogenic mechanisms responsible for AN, and particularly the implication of IgG4 autoantibodies in conduction slowing. Therapeutic anti‐CD20 monoclonal antibodies and plasma exchange have been shown to be efficient in AN. The potential of novel therapeutic strategies will be approached as well as the strengths and limitations of available autoantibody diagnosis tools. Altogether, this should bring a broad overview of the current knowledge on AN diagnosis, treatment, and physiopathology.

**Disclosure:** Nothing to disclose.

## SPS08_3

### CGRP in migraine pathophysiology

#### M. Ashina

##### Department of Neurology, Danish Headache Center, Copenhagen University Hospital‐ Rigshospitalet, Copenhagen, Denmark

Calcitonin gene–related peptide (CGRP) is now recognized as a key mediator of migraine. Released from activated trigeminal fibers, CGRP promotes vasodilation and neurogenic inflammation, setting the stage for migraine pain. The advent of oral “gepants” and monoclonal antibodies against CGRP or its receptor has revolutionized both acute and preventive migraine management, offering sustained reductions in attack frequency with favorable tolerability. This lecture will trace CGRP's journey from peptide discovery to the clinic, highlighting pivotal human models and the therapeutic breakthroughs that have reshaped migraine care.

**Disclosure:** MA is a consultant, speaker, or scientific advisor for AbbVie, Astra Zeneca, Eli Lilly, GlaxoSmithKline, Lundbeck, Pfizer, and Teva; a primary investigator for ongoing AbbVie, Lundbeck and Pfizer trials. MA reports research grants from Lundbeck Foundation and Novo Nordisk Foundation (all to institution). MA serves as associate editor of the Journal of Headache and Pain and associate editor of Brain.

## Breakthroughs in treatment Neurology—Part 2

## SPS09_1

### Rejection and approval: Lecanemab and the future of anti‐amyloid treatments

#### M. Bruno

##### Memory Clinic, Policlinico Tor Vergata, University of Rome “Tor Vergata”, Rome, Italy

The advent of monoclonal antibodies targeting amyloid‐beta represents a groundbreaking shift in Alzheimer's disease (AD) treatment, offering the first disease‐modifying therapies for early‐stage AD. While these treatments have demonstrated amyloid clearance and modest slowing of cognitive decline in clinical trials, their real‐world impact remains uncertain. Strict eligibility criteria limit patient access, with studies indicating that only a fraction of individuals with modest cognitive involvement qualify for treatment (1, 2). The EMA initially rejected lecanemab due to concerns over its clinical significance, as its modest cognitive benefits did not outweigh the risks, particularly amyloid‐related imaging abnormalities (ARIAs) (3). However, following further review and pressure from advocacy groups and clinicians, the EMA later granted approval, reflecting the ongoing debate over the balance between biological efficacy and meaningful clinical outcomes. Despite regulatory acceptance, the lack of long‐term efficacy data and the challenges of implementation in routine clinical practice further complicate the widespread adoption of these therapies. As global regulatory agencies take divergent stances on approval, the discussion continues over whether anti‐amyloid therapies truly alter the course of AD or merely offer biological rather than meaningful clinical benefits. Given the limitations of amyloid‐targeting approaches, there is increasing attention toward alternative therapeutic targets, such as tau pathology, neuroinflammation, synaptic dysfunction, and metabolic pathways. Ongoing research into tau‐targeting, anti‐inflammatory, and neuroprotective agents may offer more comprehensive and effective treatment options in the future. Further real‐world data and long‐term studies are essential to determine the role of current and emerging therapies in AD management.

**Disclosure:** Nothing to disclose.

## SPS09_2

### New concept to improve care: Rapid and early seizure termination

#### E. Trinka

##### Uniklinikum Salzburg, Christian‐Doppler‐Klinik, Department of Neurology, Salzburg, Austria

## SPS09_3

### Trial of N‐Acetyl‐L‐Leucine in Niemann‐Pick disease type C

#### M. Strupp ^1^, M. Patterson^2^, J. Raymond^2^, J. Zanrucha^2^, A. Hatcher^2^, T. Fields^2^, T. Bremova‐Ertl^3^, K. Martakis^4^

##### ^1^Department of Neurology, Hospital of the Ludwig Maximilians University, Munich, Germany; ^2^IntraBio; ^3^Department of Neurology and Center for Rare Diseases, University Hospital Inselspital Bern, Switzerland; ^4^Department of Neuropediatrics, Justus Liebig University Giessen, Germany

Introduction Niemann‐Pick disease type C (NPC) is a rare, autosomal recessive neurodegenerative disorder. The IB1001‐301 clinical trial was a Phase III, double‐blind, randomized, placebo‐controlled trial comparing N‐acetyl‐L‐leucine (NALL) with placebo for treating neurological signs and symptoms in NPC after 12 weeks with a subsequent ongoing open‐label extension phase. Methods Patients received treatment with orally administered NALL 2–3 times per day (patients 4‐12 years receiving weight‐based doses). The primary endpoint for the placebo‐controlled parent study was the Scale for the Assessment and Rating of Ataxia (SARA). Following the parent study patients could enroll into an open‐label extension phase. In the extension phase the primary endpoint was the modified 5‐Domain NPC Clinical Severity Scale (5‐Domain NPC‐CSS); comparisons were made to the expected annual trajectory of disease decline established in published natural history studies. For both endpoints a lower score represents better neurological status. Results In the parent study a cohort of 60 NPC patients aged 5‐67 years met its primary and all secondary end points. The mean (±SD) change from baseline in the SARA total score was −1.97±2.43 points after 12 weeks of receiving NALL and −0.60±2.39 points after 12 weeks of receiving placebo (least‐squares mean difference, −1.28 points; 95% confidence interval, −1.91 to −0.65; P<0.001). The results for the secondary end points were supportive of the findings in the primary analysis. The incidence of adverse events was similar with NALL and placebo, and no treatment‐related serious adverse events occurred. In the extension phase 54 patients aged 5–67 years were treated. The improvements in neurological status demonstrated in the parent study's primary SARA endpoint were sustained over the 24‐month long‐term follow‐up: the mean (±SD) change from baseline on the 5‐Domain NPC‐CSS was ‐0.24 (±2.69) on NALL, compared to +3.0 (±6.32) in the historical cohort: mean difference ‐3.24 (95% Confidence Interval (CI) ‐5.59 to ‐0.89; *p* = 0.009). NALL was well‐tolerated, and no treatment‐related serious AEs occurred. Conclusion Treatment with NALL for 12 weeks led to a significant improvement in neurological status compared to placebo. The continued treatment with NALL over 24 months was associated with a statistically significant and clinically meaningful reduction in disease progression and consistent with a neuroprotective, disease‐modifying effect.

**Disclosure:** Nothing to disclose.

## SPS09_4

### Persistent remission from treatment refractory autoimmune neuropathies by autologous CD19‐targeted CAR T cell therapy

#### J. Motte

##### Department of Neurology, St. Josef‐Hospital, Ruhr‐University Bochum, Bochum, Germany

Objective: To demonstrate the efficacy of autologous anti‐CD19 CAR T cell therapy in two severe cases of autoimmune neuropathies Methods: Two patients with a history of more than 12 months of severe tetraparesis with insufficient response to established immunotherapies were selected from our clinical cohort of autoimmune neuropathies. Patients' lymphocytes were collected by leukapheresis. CD3+ selected T cells were transduced with a second‐generation anti‐CD19 CAR construct (KYV‐101, Kyverna Therapeutics, Inc., CA, USA) resulting in CAR/KYV‐101 expression in 78 % (patient 1) and 66 % (patient 2) of T lymphocytes, respectively. Following standard lymphodepletion with fludarabine (30 mg/m^2^) and cyclophosphamide (300 mg/m^2^) CAR T cells were administered following established procedures. Results: Patients exhibited only moderate side effects after CAR T cell infusion and continuous improvement of neurological symptoms occurred starting at week 4 post CAR T cell transfer. Even without continuation of previous immunotherapies they stabilized. At 6 months post transfer they were already able to perform squats and pull‐ups or walk independently over 200 meters respectively with subsequent continuous improvement. Conclusion: Even with the availability of modern treatment approaches via anti‐FcRn antibodies or complement neutralization for chronic autoimmune neuropathies most severe disease courses still exist. CAR T cell‐based therapy may be an alternative for those patients.

**Disclosure:** JM: stock ownership from Amgen, Bayer, Sanofi, received travel grants from Alnylam, Biogen idec, Novartis AG, Teva, Eisai GmbH, Neuraxfarm, BMS and Kyverna, consulting fees from Novartis and Alnylam his research is funded by Klaus Tschira Foundation, Ruhr‐University, Bochum (FoRUM‐program); DMSG, Hertie foundation, Novartis, Kyverna, none related to this work. I declare no competing interests.

## SPS09_5

### Acetyl‐DL‐leucine in two individuals with REM sleep behavior disorder improves symptoms, reverses loss of striatal dopamine‐transporter binding and stabilizes pathological metabolic brain pattern – case reports—3‐year follow‐up

#### W. Oertel

##### Department of Neurology, Philipps University of Marburg, Marburg, Germany

Introduction: Isolated REM Sleep Behavior Disorder (iRBD) is considered a prodrome of Parkinson's disease (PD). Methods: We investigate whether the potentially disease‐modifying compound acetyl‐DL‐leucine (ADLL; 5g/d) has an effect on prodromal PD progression in 2 iRBD‐patients. Outcome parameters are RBD‐severity sum‐score (RBD‐SS‐3), dopamine‐transporter single‐photon emission computerized tomography (DAT‐SPECT) and metabolic “Parkinson‐Disease‐related‐Pattern (PDRP)”‐z‐score in 18F‐fluorodeoxyglucose positron emission tomography (FDG‐PET). Results After 3 weeks ADLL‐treatment, the RBD‐SS‐3 drops markedly in both patients and remains reduced for >18 months of ADLL‐treatment. In patient 1 (female), the DAT‐SPECT putaminal binding ratio (PBR) decreases in the 5 years pretreatment from normal (1.88) to pathological (1.22) and the patient's FDG‐PET‐PDRP‐z‐score rises from 1.72 to 3.28 (pathological). After 22 months of ADLL‐treatment, the DAT‐SPECT‐PBR increases to 1.67 and the FDG‐PET‐PDRP‐z‐score stabilizes at 3.18. Similar results are seen in patient 2 (male): his DAT‐SPECT‐PBR rises from a pretreatment value of 1.42 to 1.72 (close to normal) and the FDG‐PET‐PDRP‐z‐score decreases from 1.02 to 0.30 after 18 months of ADLL‐treatment. We will present follow‐up data of the 2 RBD patients after 2.5 to 3 years of ADLL‐therapy on the patient‐related subjective RBD‐severity score and the two objective imaging procedures DAT‐SPECT and FDG‐PET. Conclusion The results support exploration of whether ADLL may have disease‐modifying properties in prodromal PD. Oertel et al. Nature Communications 2014 Supported by ParkinsonFonds Deutschland.

**Disclosure:** Wolfgang H. Oertel has received speaker's honoria by Abbvie, the International Movement Disorders Society (IMDS) and Stada Pharma. He is a member of the advisory boards with Intrabio and MO DAG. He holds stock options with Intrabio related to this work and stock options with MODAG unrelated to this work. The institution of W.H.O.received/s scientific grants from the German Research Foundation (DFG) and the Michael J Fox Foundation unrelated to this work. Jan Booij is a consultant of GE Healthcare. The institution of J.B. received research funding from GE Healthcare. Lars Timmermann has received speaker's honoria by Abbvie, Boston Scientific, DIAPLAN, Neuraxpharma, Novartis, the IMDS and Teva. He has been a consultant for Boston Scientific. The institution of L.T. received/s funding by Boston Scientific, the DFG, the German Ministry of Education and Research, the Otto‐Loewi‐Foundation and the Deutsche Parkinson Vereinigung. Michael Strupp has received speaker's honoraria by Abbott,Auris Medical, Biogen, Eisai, Grünenthal, GSK, Henning Pharma, Interacoustics, J&J, MSD, NeuroUpdate, Otometrics, Pierre‐Fabre, TEVA, UCB, and Viatris. He acts as a consultant for Abbott, AurisMedical, Bulbitec, Heel, IntraBio, Sensorion and Vertify. He is an investor and shareholder of IntraBio. The institution of M.S. received/s support for clinical studies from Decibel, USA,Curewithin Reach, USA and Heel, Germany. The remaining authors declare no competing interests.

## SPS09_6

### Parkinson's disease in Africa; from Genome wide association to novel genetic mechanism

#### H. Holden

##### UCL Institute of Neurology, London, UK

In Parkinson's disease (PD), large‐scale genome‐wide‐association‐studies (GWAS) in European, Asian, and Latin American populations have identified multiple risk‐loci. One particular PD risk‐gene of interest is GBA1 gene which encodes glucocerebrosidase (GCase).

Until recently, PD in African populations remain completely unexplored. We performed a comprehensive genome‐wide‐assessment of PD in 197,918 individuals (1,488 cases; 196,430 controls) of African (Nigerian) and African‐admixed ancestry, characterizing population‐specific risk, coding and structural genetic variation. We identified a novel common risk‐factor for PD and age‐at‐onset at the GBA1 locus (risk, rs3115534T>G; OR = 1.58, 95% CI = 1.37‐1.80, *p* = 2.397E‐14). Sequencing did not reveal any coding/structural variation. However, we identified GBA1 rs3115534T/G signal mediates PD risk via expression quantitative‐trait locus (eQTL) mechanisms, found to be extremely rare in non‐African populations.

Using full‐length RNA transcript sequencing, we identified partial intron‐8 expression in risk variant carriers (G) but not in nonvariant carriers (T). Clustered regularly interspaced short palindromic repeats editing of the reported index variant (rs3115534) revealed that this sequence alteration is responsible for driving the production of transcripts containing intron‐8. We showed the variant is a key intronic branchpoint sequence and measuring glucocerebrosidase activity, we identified a dose‐dependent reduction in risk variant carriers, a potential therapeutic target in an underserved and underrepresented population.

**Disclosure:** Nothing to disclose.

## Condensed Autogenic Training Session (Part 2)

## SPS11_1

### Condensed autogenic training session

#### M. Hilz

##### University of Erlangen‐Nuremberg, Germany, and Icahn School of Medicine at Mt. Sinai, New York, USA

This session recapitulates the Autogenic Training (AT) concept and practices AT. Participants in the first session can deepen their AT experiences, newcomers may start perceiving relaxing AT effects. AT is practiced in a quiet environment. Participants should join with “an empty bladder and an open mind”. They shall turn off phones, remove watches, glasses, wear comfortable clothes, and loosen tight clothing. AT is practiced in a comfortable, sitting, reclined, or lying position. AT‐participants should internalize the trainer's hetero‐suggestions and repeatedly self‐suggest the proposed sensations. They can close their eyes but shall not fall asleep. The initial suggestion conveys calm and resilience against any disturbances. Then follow suggestions of heaviness and afterwards warmth of the arms and legs. The subsequent suggestion “the forehead is comfortably cool” helps avoid unpleasant sensations of a warm or hot head potentially associated with hotheadedness. Of note, cold‐face stimulation induces parasympathetic activation. The next suggestion of a calm, comfortable, rhythmic, and regular heartbeat furthers relaxation. Then follows the self‐suggestion of free, automatic, unrestricted, and comfortable breathing. Slow breathing again augments cardiovagal activity and thus relaxation. The so‐called “solar plexus exercise” suggests that the upper abdominal or epigastric area with its large vasculature and autonomic nerve plexus is pleasantly warm and well‐perfused. There are additional suggestions such as warmth and relaxation of the neck and shoulders, or individual formulas. Participants may end AT by actively reestablishing muscle tone and full alertness. Consequent AT reduces negative stress effects and mitigates somatic or mental complaints.

**Disclosure:** I have nothing to disclose related to this presentation. I received lecturing honoraria from Sanofi and travel support from Sanofi and Amicus Therapeutics.

